# Community-Acquired Methicillin-Resistant *Staphylococcus aureus* Prostatic Abscess Presenting as Acute Urinary Retention: A Case Report and Review of the Literature

**DOI:** 10.1155/2013/761793

**Published:** 2013-05-12

**Authors:** Ali Naboush, Ali Abou Yassine, Mohamad Yasmin, Neville Mobarakai

**Affiliations:** ^1^Department of Internal Medicine, Staten Island University Hospital, 475 Seaview Avenue, Staten Island, NY 10305, USA; ^2^Department of Infectious Diseases, Staten Island University Hospital, 475 Seaview Avenue, Staten Island, NY 10305, USA

## Abstract

*Background*. Community-associated MRSA (CA-MRSA) strains have emerged as a substantial cause of infection in individuals without exposure to the healthcare system. Prostatic abscess is an uncommon disease. To date, there are only 6 published reports of a prostatic abscess secondary to CA-MRSA. *Case Description*. A 52-year-old diabetic Caucasian presented to the emergency department with severe lower abdominal pain of few hours duration, urinary frequency, and dribbling over the last 3 weeks. Physical examination was remarkable for an enlarged nontender prostate. A urine analysis showed pyuria while urine cultures grew CA-MRSA. Computed tomography of the abdomen and pelvis showed multiple prostate abscesses and a thickened urinary bladder wall. A TURP was performed by the urology team and pathology showed severe acute and chronic prostatitis with abscess formation and necrotic tissue. Our treatment regimen included IV vancomycin followed by oral trimethoprim/sulfamethoxazole and rifampin. Eradication of CA-MRSA was confirmed by follow-up cultures 2 months following discharge. *Conclusion*. This case illustrates the successful identification, diagnosis, and prompt treatment of a prostatic abscess secondary to CA-MRSA in a diabetic patient without recent hospitalization. Early treatment with antibiotics and transurethral resection of the prostate abscess led to a shortened hospital stay and decreased morbidity.

## 1. Introduction

*Staphylococcus* (*S.*) *aureus*-induced infections have rapidly increased during the last decade with methicillin-resistant *S. aureus* (MRSA) currently accounting for >50% of staphylococcal disease [1]. Although formerly considered to be an organism solely limited to healthcare contact, community-associated MRSA (CA-MRSA) strains have emerged as a substantial cause of infection in individuals without exposure to the healthcare system [2]. CA-MRSA has in fact recently emerged as the predominant cause of MRSA disease [3]. 

Prostatic abscess is an uncommon disease because of the wide use of broad-spectrum antibiotics in patients with lower urinary tract symptoms (LUTS) [4]. It is primarily identified in patients with preexisting medical conditions, chronic indwelling catheters, instrumentation of the lower genitourinary tract, diabetes mellitus (DM), human immunodeficiency virus (HIV) infection, chronic hemodialysis requirements, and other causes of compromised immunity [5]. Prostatic abscess formation which was primarily caused by *Neisseria gonorrhoeae* (75% of cases) in the preantibiotic era is now predominantly caused by *E. coli *(antibiotic era/60% to 80% of cases) [4]. Other significant pathogens include *Pseudomonas *species, *Staphylococcus *species, and occasionally obligate anaerobic bacteria [6]. With the increased incidence of CA-MRSA, several reports emerged in the literature depicting prostatic abscess development mainly within immunosuppressed patients [7–12]. In this report, we describe a case of CA-MRSA prostatic abscess in a diabetic patient presenting with acute urinary retention.

## 2. Case Report

**Figure 1:**
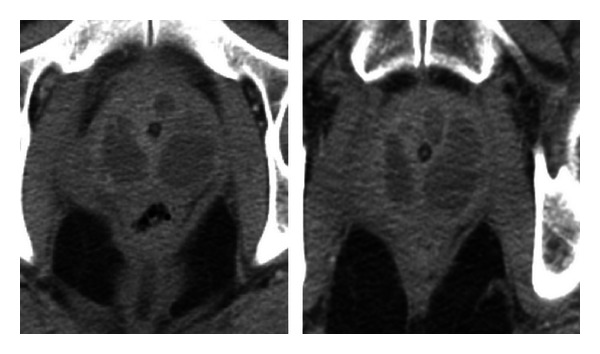
Showing the multiple prostatic abscesses on abdominopelvical CT.

A 52-year-old Caucasian male presented to the emergency department (ED) for severe lower abdominal pain of few hours duration. He is known to have a history of uncontrolled DM type 2 for the last 20 years complicated by severe peripheral neuropathy, right diabetic foot ulcer (treated adequately more than a year ago), and peripheral vascular disease (PVD). The patient had been complaining of frequency and dribbling for the last 3 weeks. He denied any dysuria, hematuria, fever, or chills. Physical examination was remarkable for an enlarged nontender prostate. A urethral catheter was inserted yielding 500 cc of clear yellow nonturbid urine. A urine analysis showed pyuria. A specimen of urine was sent for culture. The patient was discharged on a cephalosporin. Two days later, he was readmitted with severe abdominal pain and a urethral catheter yielded 750 cc of urine. He was hemodynamically stable with no evidence of sepsis. Urine culture grew MRSA (sensitive to all antibiotics except oxacillin and ciprofloxacin). Blood work demonstrated hyperglycemia, anemia of chronic disease, and hypoalbuminemia. Further lab work showed a glycosylated hemoglobin (A1c) of 14.7 and erythrocyte sedimentation rate (ESR) of 122. The patient was started on vancomycin intravenously (IV). Computed tomography (CT) of the abdomen and pelvis showed multiple prostate abscesses, the largest measuring 4.6 × 2.4 × 3.5 cm, and a thickened urinary bladder wall (Figure 1). Urology service performed a transurethral unroofing of the abscess. The pathology report later showed severe acute and chronic prostatitis with abscess formation, necrotic tissue focally with overgrowth of bacteria. Our patient received IV vancomycin for a total of 5 days and was thereafter discharged on a 3-week combination of trimethoprim/sulfamethoxazole (TMX)/rifampin followed by 1 week of TMX. The patient was followed at the urology clinics where his urethral catheter was removed and eradication of CA-MRSA was confirmed by follow-up cultures 2 months following discharge.

## 3. Discussion

Prostatic abscess is an uncommon disease because of the widespread use of broad-spectrum antibiotics in patients with LUTS. It is primarily identified in patients with certain preexisting medical conditions (DM, HIV infection, and chronic hemodialysis requirement), chronic indwelling catheters, instrumentation of the lower genitourinary tract, and other causes of compromised immunity. CA-MRSA as a cause of prostatic abscess is quite unusual with 6 reported cases (see Table 1). The majority of CA-MRSA prostatic abscess patients in the literature presented with hesitancy, weakened stream, dysuria, fever, perianal discomfort, tender prostate, and leukocytosis. In contrast, our patient presented with symptoms of benign prostatic hyperplasia (BPH) rather than infection. Prostatic abscess is difficult to diagnose because clinical presentations may mimic those of lower UTI [6], or BPH as in our case. In our patient, the absence of BPH and systemic signs of infection coupled with a positive urine culture for CA-MRSA raise the suspicion for an abscess. Furthermore, a rise in inflammatory markers like CRP and ESR increases the likelihood of an underlying chronic prostatitis/abscess formation. That being mentioned, the diagnosis of a prostatic abscess remains a challenge for physicians due to its rarity along with the lack of a gold diagnostic standard [13].

Most (5 out of 7) of the patients diagnosed with a prostatic abscess including ours had a history of uncontrolled DM. Of the remaining two patients in the literature, one had AIDS while the other had chronic hepatitis C and IV drug abuse. Thus, an immuno compromised state seems to be a common risk factor for the formation of CA-MRSA prostatic abscess. Other known risk factors include indwelling catheters, a recent prostate biopsy, and instrumentation of the lower urinary tract [14]. 

It is thought that most CA-MRSA infections spread through the hematogenous route from the skin (furuncles, abrasions) given that CA-MRSA comprises a large percentage of skin and soft tissue infections in patients within emergency departments [3]. Indeed hematogenous spread seems to be a plausible mechanism bearing in mind that only 2 reported cases had positive urine cultures. Another likely scenario could involve bacterial translocation from the perineal skin to the urine leading to acute prostatitis and subsequent abscess formation.

The diagnosis of CA-MRSA prostatic abscess is based on the definition of MRSA infection occurring in the community [3]. This characterization is however extended to include the initial 48 hours of hospitalization in patients with no history of admission to a hospital/nursing home in the past year and no history of dialysis, surgery, permanent indwelling catheters, or medical devices that pass through the skin to the body [15]. Our case report as well as the other six mentioned cases had all the criteria defining CA-MRSA described above. The pathology report in our case described necrosis of the prostate: a well-known characteristic virulence feature of CA-MRSA USA300 clone [16]. 

The best imaging modality to detect a prostatic abscess is a CT of the abdomen and pelvic area. A CT scan compared to TRUS provides better characterization of a lesion, detects spread beyond its borders, and aids in delineating an abscess from neoplastic processes, cystic lesions, or granulomas [17]. 

Prompt medical and surgical management of CA-MRSA prostatic abscess is crucial to prevent progression to sepsis and death [10]. However, the lack of clear standards and guidelines makes this difficult. The proposed treatment is probably a week of IV vancomycin followed by 4 weeks of TMX/rifampin or a combination of both but there are no clear guidelines [13]. Our patient was treated with IV vancomycin for 5 days followed by a 3-week combination of TMX/rifampin and a 1-week course of TMX. Surgical drainage is mandatory for abscesses more than 1 cm or those refractory to conservative treatment. Transurethral resection of the prostatic abscess (TURP) may be the procedure of choice [18]. However, a less invasive approach to manage the abscess would be a transrectal-ultrasound- (TRUS-) guided needle aspiration [19]. A review of the limited literature available demonstrated that patients treated by TURP did not develop infection recurrence or mortality. In that respect one can conclude that TURP-treated patients had a more favorable outcome. On the other hand, patients treated with needle aspiration resulted in a longer hospitalization and high recurrence [20]. Our patient was successfully treated with antibiotics and the TURP approach. His hospitalization course was uncomplicated and short. To date he has not had a recurrence of his infection.

## 4. Conclusion

This case is unusual in that it is the first case of CA-MRSA prostatic abscess to present as urinary retention mimicking BPH without systemic signs of infection. The identification, diagnosis, and prompt treatment of a prostatic abscess secondary to CA-MRSA remain a challenge. Early treatment with antibiotics and transurethral resection of the prostate abscess will lead to a shortened hospital stay, prevention of recurrence and mortality.

## Figures and Tables

**Table 1:** Summarizing the cases of CA-MRSA prostatic abscess reported.

Author year	Age	Clinical findings	Comorbidites	Drainage method	Microbiology	Outcome	Imaging	Country of origin
Baker et al. 2004 [8]	43	Hesitancy, low stream, dysuria, afebrile, and leukocytosis	IVD abuser, hep C	TURP 3 weeks of IV vancomycin followed by 2 weeks of TMX	CA-MRSA in prostatic fluid	Survived	Right lobe 4.4 × 2.7 cm	USA

Pierce et al. 2008 [12]	64	Dysuria, tender prostate, fever, and leukocytosis	Newly diagnosed diabetes	Percutaneous drainage Vancomycin IV for 4 weeks	CA-MRSA in blood, urine, and prostatic fluid	Survived	Prostatic abscess 10 × 6 cm	USA

Gautam et al. 2008 [10]	51	Fever, dysuria, and suprapubic pain	Newly diagnosed AIDS	TURP Vancomycin TMX, ciprofloxacin	CA-MRSA in prostatic fluid	Expired	Multiple low echogenic areas suggestive of an abscess	USA

Abreu et al. 2011 [7]	59	Fever, dysuria, frequency, weak urinary stream, leukocytosis, and tender prostate	Diabetes Suppurative lesions in both nasal cavities.	Percutaneous drainage Vancomycin IV followed by po TMX	CA-MRSA in blood and prostatic fluid	Survived	Hypodense areas bilaterally; both seminal vesicles were distended	Uruguay

Park et al. 2011 [11]	45	Dysuria, perianal discomfort, fever, and leukocytosis	Diabetes	TURP+ vancomycin for 10 days	CA-MRSA in prostatic fluid	Survived	Large abscess, measuring 7 × 10 cm, bilateral, and extended into both seminal vesicles	Republic of Korea

Flannery and Humphrey 2012 [9]	49	Difficult urination, micturition, afebrile, and leukocytosis	Diabetes	TURP+ vancomycin Doxycycline (1 month)	CA-MRSA in urine and prostatic fluid	Survived	Hypodensities consistent with prostate abscesses	USA

This report 2013	52	Acute urinary retention, afebrile, and no leukocytosis	Diabetes	TURP+ vancomycin 5 days TMX, rifampin (1 month)	CA-MRSA in urine, blood, and prostatic fluid	Survived	Multiple prostate abscesses, the largest measuring 4.6 × 2.4 × 3.5 cm, and a thickened urinary bladder wall	USA

## Abbreviations

MRSA: Methicillin-resistant *Staphylococcus aureus *
CA-MRSA: Community-associated MRSA
TURP: Transurethral resection of the prostate
LUTS: Lower urinary tract symptoms
BPH: Benign prostatic hyperplasia
TMX: Trimethoprim/sulfamethoxazole.
